# Extracellular vesicles: the next generation of biomarkers for liquid biopsy-based prostate cancer diagnosis

**DOI:** 10.7150/thno.39486

**Published:** 2020-01-16

**Authors:** Bairen Pang, Ying Zhu, Jie Ni, James Thompson, David Malouf, Joseph Bucci, Peter Graham, Yong Li

**Affiliations:** 1 St George and Sutherland Clinical School, Faculty of Medicine, University of New South Wales, Sydney, NSW 2052, Australia; 2 Cancer Care Centre, St. George Hospital, Sydney, NSW 2217, Australia; 3 Department of Urology, St George Hospital, Sydney, NSW 2217, Australia; 4 Garvan Institute of Medical Research/ APCRC, Sydney, UNSW, 2010, Australia; 5 School of Basic Medical Sciences, Zhengzhou University, Henan 450001, China

**Keywords:** prostate cancer, extracellular vesicles, biomarker, liquid biopsy, diagnosis

## Abstract

Prostate cancer (PCa) is a leading cause of cancer death for males in western countries. The current gold standard for PCa diagnosis - template needle biopsies - often does not convey a true representation of the molecular profile given sampling error and complex tumour heterogeneity. Presently available biomarker blood tests have limited accuracy. There is a growing demand for novel diagnostic approaches to reduce both the number of men with an abnormal PSA/ DRE who undergo invasive biopsy and the number of cores collected per biopsy. 'Liquid biopsy' is a minimally invasive biofluid-based approach that has the potential to provide information and improve the accuracy of diagnosis for patients' treatment selection, prognostic counselling and development of risk-adjusted follow-up protocols. Extracellular vesicles (EVs) are lipid bilayer-delimited particles released by tumour cells which may provide a real-time snapshot of the entire tumour in a non-invasive way. EVs can regulate physiological processes and mediate systemic dissemination of various types of cancers. Emerging evidence suggests that EVs have crucial roles in PCa development and metastasis. Most importantly, EVs are directly derived from their parent cells with their information. EVs contain components including proteins, mRNAs, DNA fragments, non-coding RNAs and lipids, and play a critical role in intercellular communication. Therefore, EVs hold promise for the discovery of liquid biopsy-based biomarkers for PCa diagnosis. Here, we review the current approaches for EV isolation and analysis, summarise the recent advances in EV protein biomarkers in PCa and focus on liquid biopsy-based EV biomarkers in PCa diagnosis for personalised medicine.

## Introduction

Prostate cancer (PCa) is the most common solid-organ cancer in men and the second most common cause of cancer death in men in western countries. The American Cancer Society reported 164,690 new cases of PCa in 2018 in males and the lifetime risk of PCa is approximately 1 in 9 in the United States 1. Most cases of PCa occur in older men (age above 65 years); only 10% of newly diagnosed PCa in the USA occurs in men under 55, however this younger group has recently gained attention due to a rise in the number of cases and the higher impact of PCa on mortality and quality-adjusted life years lost in young men 2.

The current standard biomarker in blood testing, prostate specific antigen (PSA), has proven controversial as a screening tool and the harms may outweigh the benefits for asymptomatic men, because of its high false-positive rate 3, 4. PSA cannot reliably differentiate between benign prostate conditions [prostatitis and benign prostatic hyperplasia (BPH)], indolent cancers (unlikely to cause symptoms or death in a man's lifetime) and aggressive/ advanced cancers that will likely cause symptoms and/or death if not treated promptly. Studies indicate that PSA screening leads to over-diagnosis and over-treatment in 20% to 67% of cancer cases 3, 5. Moreover, PCa still occasionally presents with symptoms of metastases, with or without PSA screening 6. All these imply that current screening and treatment tools may do more harm than good when combining the harms associated with a false positive or negative PSA test, unnecessary biopsies and unnecessary over-treatment, all for a modest survival gain 7. Therefore, novel biomarkers with higher cancer specificity to replace or complement PSA are in a great demand for earlier detection and better risk stratification to guide treatment selection 3, 8, 9.

In men with an abnormal PSA or DRE, the current gold standard to confirm or exclude PCa is a 12-30 core template needle biopsy via a transrectal route. This is associated with risks of anaesthesia, post-biopsy septicaemia, bleeding, urinary retention, pain, psychological distress and sexual dysfunction for patients 10, 11. Therefore, there is a growing demand for new approaches to reduce the number of unnecessary biopsies and the number of cores collected. In addition, the main limitation of needle biopsies is sampling error due to a high level of tumour multi-focality and genomic heterogeneity (both within each patient's prostate and between patients) which is typical for PCa 12, 13. Studies indicate that PCa may originate from both luminal and basal epithelial cells 14-17. However, current diagnostic methods solely rely on arbitrary architectural anomalies on histology such as using the *absence* of basal cells around glands to define prostate adenocarcinoma. Consequently, the significance of basal-cell differentiation in PCa diagnosis is excessively underestimated. These limitations of biopsy histology may be addressed by complementary liquid biopsy testing.

Extracellular vesicles (EVs) are particles released from nearly all kinds of cells that are delimited by a lipid bilayer and cannot replicate. EVs were under-appreciated as “cell dust” for many years 18 and have brought to people's attention recently. A major breakthrough which inspired broader research in this field was the discovery that EVs play a significant role in intercellular communication 19-21. The mechanisms of biogenesis and recruitment of cargo therein under this complex communication system are not yet fully understood, but studies have already demonstrated that EVs can transport cargos of proteins, RNAs and lipids and modulate target cells 20, 22. Thus, EVs can be considered as an all-in-one complex biomarker. This is important for cancer diagnosis because EVs provide a platform to combine individual molecules (e.g. mRNAs, non-coding RNAs, lipids and proteins) into an integrated multi-faceted “omics” tumour profile thus providing information that cannot be obtained by the needle biopsy alone. Research also suggests that EVs can regulate physiological processes and mediate systemic dissemination of various cancers 23. Therefore, EVs hold promise for the discovery of new liquid biopsy-based biomarkers for PCa diagnosis and monitoring. Due to the challenges of current PCa diagnosis mentioned above, there is an unmet demand for applying EVs as a promising approach to complement PSA, biopsy and novel diagnostic imaging tools such as multi-parametric MRI and Gallium-68 prostate-specific membrane antigen (PSMA) PET-CT scans. Several studies have compared the body fluids from PCa patients and control subjects, indicating that the EV cargo is representative for the parental cells and the conditions in which they are produced 24-26. Thus, EVs demonstrate a promising source for PCa diagnosis.

This article reviews current approaches for EV isolation and analysis including conventional and novel methods, summarises EV-based protein biomarkers used in PCa, and gives some typical examples on recent works on how EV biomarkers applied in liquid biopsy-based PCa diagnosis and monitoring, aiming towards the development of personalised medicine. Furthermore, we discuss the current difficulties in EV isolation, especially for plasma samples. Finally, we hope this review can shed some light on future directions in this field for PCa diagnosis.

## Liquid biopsy in prostate cancer

A liquid biopsy refers to using a biofluid sample such as blood, urine, cerebrospinal fluid and seminal plasma to detect and analyse biological markers to evaluate disease and determine PCa treatment options. This contrasts with needle biopsy in PCa, of which a small portion of tumour samples is removed from the prostate gland and analysed with histopathology. Normally, needle cores are performed using a core-biopsy gun for large pieces of tissue (up to 23mm/core) and 12, 18 or 24 samples per participant may be taken due to the heterogeneity of tumour, which may cause pain, bleeding, sexual dysfunction, frequency and urgency of urination, acute urinary retention or even life-threatening septicaemia in up to 2% of cases 27. In this sense, liquid biopsy has many merits. Firstly, it is a non-invasive way which solves the issues of invasiveness and limited samples with tissue biopsy; Secondly, as the biomarkers are homogeneously circulating in the body fluid, they capture the entire heterogeneity of cancer and can monitor tumour changes in real-time; Lastly, the analysis of circulating biomarkers can be performed in a fast and high-throughput way, which is essential in a clinical setting so that more accurate therapeutic strategies can be determined in-time.

**Figure 1:**
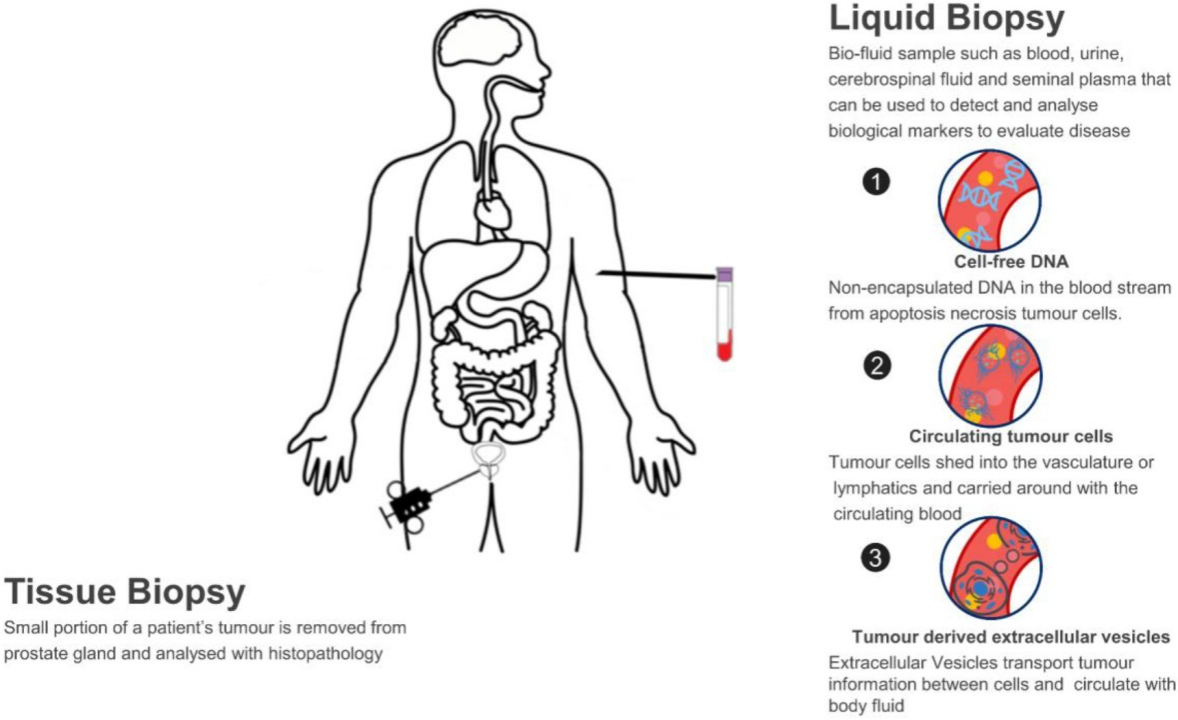
Liquid biopsy as a non-invasive novel technique for PCa diagnosis.

Several types of biomarkers can be used in liquid biopsies: proteins such as PSA, circulating tumour cells (CTCs), cell-free DNA (cfDNA) and EVs which are new types of biomarkers studied recently (**Figure 1**). Compared to CTCs, EVs are significantly more abundant in plasma/serum (10^9-12^/mL) 28-31. Comparing with cfDNA, EVs are much more stable in circulation as their contents are well-protected within a lipid membrane 32. Instead of a single biomarker, EVs carry various information cargo, such as proteins, nuclear acids, lipids and metabolites from parent cells and have the capability for information exchange. It was previously believed that only molecules released from cells and acting in a systemic, autocrine or paracrine manner participate in mediating intercellular communication. However, things changed when EVs were found in fusion with or budding from plasma membrane. Actually, EVs are able to affect the neighbouring cells in different ways, for example by transferring various molecules or by inducing intracellular signalling pathways 20, 33. Furthermore, EVs are considered promising in cancer research due to their carriage of tumour-related molecules and their role in cancer cell survival, invasiveness and metastases 34-36. Researchers have also shown that biofluid samples from prostate, ovarian and lung cancer patients have higher levels of EVs compared to control subjects, providing further evidence of their clinical significance 37-39.

## EVs isolation and detection in prostate cancer

**Figure 2:**
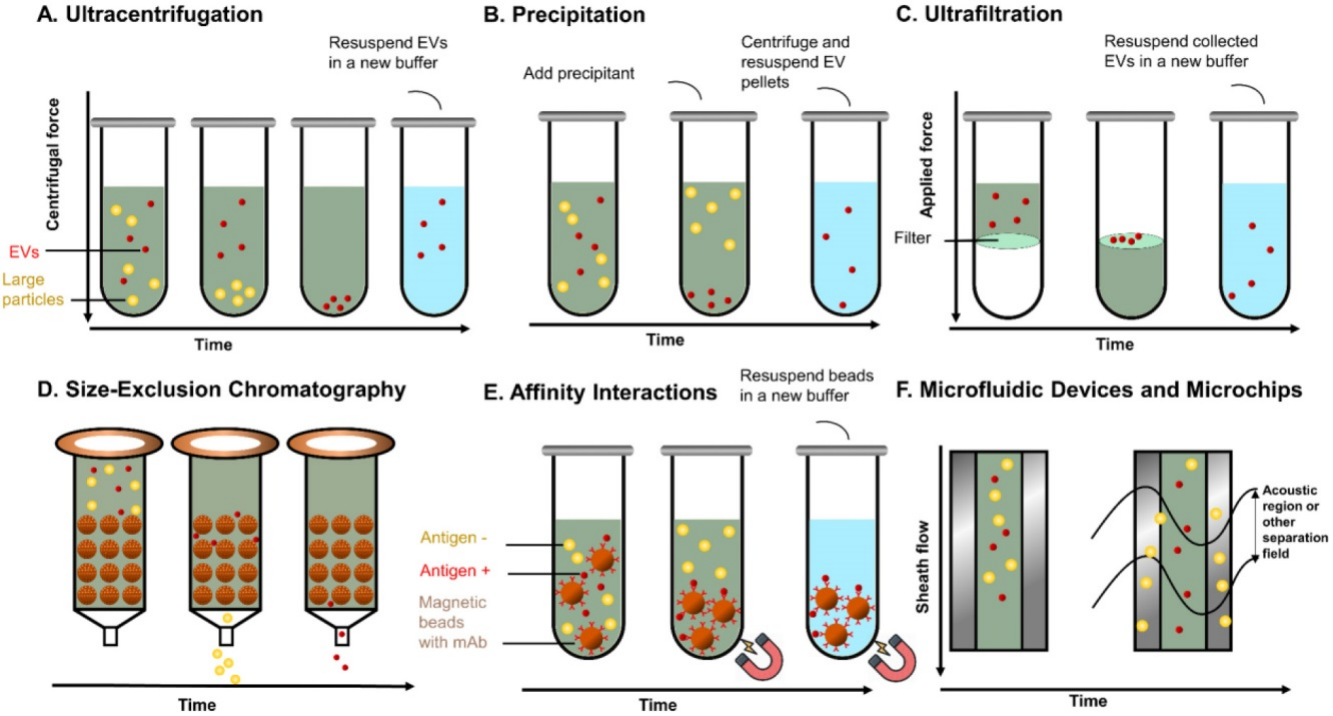
Principles of common EVs isolating methods. (A) UC separates EVs based on sedimentation speed. Large non-EV particles (yellow) with a higher density are collected earlier at the bottom. EVs (red) are collected with a higher centrifuge force with a buffer (blue) replacement to remove soluble contaminates. (B) Precipitation reagent is added to change the solubility of EVs and soluble proteins, so that EVs can be separated with lower speed centrifugation. (C) UF separates EVs with specific cut-off membranes. Soluble proteins and particles smaller than EVs are pushed through the membrane, whereas EVs are collected on the membrane. (D) In SEC, particles with different sizes pass the column with different speeds by compressing through porous matrix (brown ball), so that EVs and particles with different sizes come out in different fractions. (E) Immunoaffinity magnetic beads are added to the EVs mixtures and capture EVs. The coated monoclonal antibody on the bead's surface binds to an antigen exposed on the targeted (red) EVs only. Beads with captured EVs are separated by the magnet, whereas non-EVs particles are washed out. (F). Field-based microfluidic device separates EVs thorough microchannels along the flow direction using external forces (such as surface acoustic wave) or physical obstacles [such as nanoscale deterministic lateral displacement (nanoDLD)]. Large particles are deviated towards one side, while small EVs are deviated towards another side.

EVs can be considered as an all-in-one complex biomarker and a standout among various types of liquid biopsy, but their isolation and analysis are quite challenging because of their small sizes and low densities. Many commercial products and new techniques have been developed for the isolation of EVs from different biological fluids, aiming towards improving the recovery (yield) and specificity (purity). The populations of EVs are heterogeneous with variations in size distribution from typically 30 nm to 1000 nm as well as other physical properties including mass density and shape. Other than physical properties, differences in biochemical and physicochemical properties, such as solubilities, surface identity (proteins), charge, and hydrodynamic property are also being taken into consideration for isolating EVs and their subpopulations. Since EVs broadly exist in mammal biofluids, in which case each biological fluid presents specific biochemical and physical characteristics, it requires different isolation and purification processes. EVs and their subpopulations can be segregated to various extents using different methods or their combinations. Here, we discuss the common isolation methods and novel approaches of each method in PCa research in this section. The commonly used EV isolation methods in PCa are summarised in **Table 1** and a schematic diagram outlining the various isolation principles is shown in **Figure 2**.

## Ultracentrifugation and density gradients

Ultracentrifugation (UC) is presently the gold standard method for EV isolation. Current EV protein biomarker studies in PCa mainly focus on UC-based isolation methods, which can be seen from **Table 2**. The separation principle of this method is based on the sedimentation speed difference between EVs and other particles. UC has large sample capacity and low following cost. One stand out point is that UC can generate an EV pellet while most other methods maintain EVs within a solvent. This may be preferable for downstream analysis as it provides a richer concentration of EVs without being diluted by the buffer. Pellets of PCa EVs isolated by UC from the urine have been studied for miRNA profiling with good performance (using miR-19b versus miR-16 with 100%/93% and 95%/79% specificity/sensitivity) to distinguish PCa from healthy individuals 25. UC can be used inclusive of density gradients to further separate EV subpopulations and remove contaminating proteins. Along with the combination of density gradient with UC, EV purity and the efficiency of particle separation are increased according to their buoyant density for specific populations of EVs 40, 41, 43. Sucrose-gradients or iso-osmotic gradients can be used to capture exosomes (endosome-originated EVs) or exosome-like particles with similar density between 1.1 and 1.19g/mL. Several studies have shown that iso-osmotic gradients give better results than sucrose, since its property can form various densities of isosmotic solutions which maintain the vesicle physical properties and protein contents 40, 42, as well as allow segmentation of the EVs from virions 44.

Despite being the most commonly used approach for isolating EVs, UC has several limitations. EVs are nanoparticles with extremely close density to protein aggregates, apoptotic bodies and other non-EVs particles that may present in the EV fraction obtained by UC. Different subtypes of EVs such as those with different sizes may also sediment together during UC. Numerous recent studies have shown that the unexpected aggregation during the UC isolation process may lead to erroneous interpretation and interfere with EV surface antigen detection on subsequent analysis 67-70. Lengthy processing time, contamination with non-EV proteins and high capital costs also restrict the use of UC for EV isolation. Furthermore, Momen-Heravi *et al.* 71 reported that viscosity has a significant effect on EV recovery. Biofluids with higher viscosity such as plasma and serum demonstrated lower sedimentation efficiency 71. Thus, UC is considered less applicable in a clinical setting wherein blood is the main biofluid collected. As density gradient centrifugation brings more work burden and complexity than UC, it is also impractical in a high-throughput commercial setting.

## Precipitation

Precipitation of EVs could be less time-consuming compared to UC by adding precipitation reagent and isolating EVs with lower centrifugal forces. The precipitation reagent can be hydrophilic polymers (e.g. polyethylene glycols), protamine, sodium acetate or event organic solvent which initiates hydrophobic interactions and thus precipitates EVs or proteins based on solubility change 46-49. There are many commercial kits (e.g., Total Exosome Isolation Reagent, ExoQuick) based on precipitation. The procedure is usually simple, fast, scalable and most importantly does not deform EVs. Van *et al*. 42 have indicated that the amount of EVs and their characteristic proteins and miRNAs using precipitation reagent are obviously larger compared to UC. A recent study showed that ExoQuick precipitation solution (System Biosciences) and Total Exosome Isolation kit (Invitrogen) have a higher total protein amount and more efficient (less plasma amount and processing time) for EV isolation from PCa plasma samples compared to UC 50. Between them, ExoQuick precipitation showed a better performance in comparing the EVs markers (CD63, CD9, CD81), protein content (bicinchoninic acid assay) to the UC and the Invitrogen kit. However, EVs isolated by ExoQuick have more interference with the electron beam, thus cannot be used directly for transmission electron microscopy whereas EVs isolated from Invitrogen kit and UC can be used directly. These indicate that precipitation method might obtain contaminants such as protein complex, RNA complex, viral and reagent residual which limit it as a standalone EV isolation method in clinical research. Lionel *et al.* 51 also suggest that EVs have natural affinity with immunoglobulins and thus are easily co-precipitated with them by precipitation methods. The study performed by Natasa *et al*. 45 demonstrated that plasma samples loaded with commercial precipitation kits derive pellets with major protein complexes instead of EVs. To conclude, current precipitation-based methods yield low EVs purity with high contaminates from both original biofluids and precipitation reagents, which limits their use in clinical research. Further substantive EV characterizing are highly recommended before following EVs studies for this method. Combination of the precipitation with other methods such as immunoaffinity capture is highly recommended to improve the purity. Precipitation may be more suitable for cell culture media than human samples due to the relevant simpler media environment. Moreover, these commercial kits can be expensive for large-scale usage.

## Ultrafiltration

Ultrafiltration (UF) is designed to separate EVs by applying membrane filters with appropriate pores size. Thus, particles with a specific size are concentrated on the membrane. Designed molecular weight cut-off (MWCO) of the membrane can be used to either remove or enrich particles with different molecular weight. For instance, Willms *et al.* 72 isolated EVs by combining size-exclusion chromatography (SEC) with both 100 kDa and 10 kDa MWCO UF membrane for removal and enrichment purposes to keep EVs integrity compared to UC. UF is especially useful for processing large volume sample such as urine, thus makes it particularly useful for urine-based EV study for PCa. However, it doesn't mean UF can be only used for large volume sample. Different types of membrane have been investigated and many of them are commercially available. For instance, Cheruvanky *et al*. 53 used nanomembrane concentrators (Sartorius, Germany) for a rapid isolation of EVs from the urinary sample, which can be used to enrich exosomal proteins with low sample volume 53. Another study by Bryzgunova *et al.* demonstrated that small EVs (based on size ranging 30-100 nm) isolated from both healthy donors and PCa patients count to 95% and 90% of total EVs respectively using 100 nm UF membrane, and the isolated samples are positive to EV biomarkers CD63, CD9 and CD24 25. Recently, a novel filtration-based EV isolation tool, the Exosome Total Isolation Chip (ExoTIC), was developed by Liu *et al*. 52. Utilizing a nanoporous membrane, clinical samples (such as plasma, urine, and lavage) were passed through to isolate and enrich EVs in the 30-200 nm size range. ExoTIC has shown an EV yield 4-1000 fold higher compared to UC from biofluids (plasma, urine, and lavage). Specifically, ExoTIC was observed to provide a higher expression of certain miRNAs (such as hsa-miR-1246, hsa-miR-134) in lung cancer cell lines (HCC827 and H1650) compared to UC. Meanwhile, EV protein expression from PCa cell line (22Rv1) was higher as well, with 29% of identified EV proteins contributing to UC and 62-75% contributing to ExoTIC, respectively. These results suggest that UF can efficiently isolate vesicles with specific particles size and may be suitable for isolating specific EV subtypes of miRNAs compared to UC.

UF is considered a safer and more time-efficient method by avoiding excessive centrifuge steps. However, applying UF may cause reduction in EVs quantity and EVs related proteins. Cheruvanky *et al*. 53 indicated that exosomal markers such as neuron-specific enolase (NSE), annexin V and podocalyxin (PODXL) can be readily recovered from urine samples using UF. However, some other EV proteins such as tumour susceptibility gene 101 (TSG101) and aquaporin-2 (AQP2) are likely to adhere to the UF membrane result in a low recovery from the retentate. Furthermore, Busatto *el al.* 57 and Rood *et al.* 54 reported that EVs isolated with UF have excessive protein contamination such as albumin and other soluble proteins.

## Fractionation

UF systems can either be dead-end flow or cross-flow in which the feed is passed through a bed or tangentially across the filter surface. Traditional dead-end flow can combine with SEC or other methods to purify the fraction with batch processes 72, 73 and no extra energy needs to be introduced. In dead-end filtration, larger particles may clog the membrane and reduce the separation efficiency. Cross-flow UF is considered to be more efficient for continuous operation compared to dead-end flow owing to the continuous flush of the membrane surface. Recently, application of cross-flow filtrations, such as field flow fractionation (FFF), asymmetrical flow field-flow fractionation (AF4) and tangential flow filtration (TFF) has been introduced to the EV separation. These systematic filtration or fractionation methods can be combined with other modern techniques for further analysis. Yang *et al*. 56 used FFF to isolate exosomes from PCa urine sample and further carried out lipidomic analysis using nanoflow ultrahigh performance liquid chromatography-electrospray ionization-tandem mass spectrometry. Their results showed that there is a decrease in neutral lipids such as diacylglycerol and triacylglycerol but an increase in total lipids when comparing PCa patients to healthy controls. Using the state-of-the-art AF4, Zhang *et al*. 55 recently reported two exosome subtypes (large exosome vesicles, Exo-L, 90-120 nm; small exosome vesicles, Exo-S, 60-80 nm). They identified as well a previously unknown population with non-membranous structure (termed “exomeres”, ~35 nm). Each of the subpopulations has unique proteins, lipids and nuclear acid profiles and biophysical properties. This study demonstrated for the first time that AF4 is capable to provide EVs subpopulation to some extent based on size, which may open a new avenue for more specific classification of EVs. However, the robustness of this system and its capability for EVs classification need further validation from other studies. TFF was shown to be able to process large scalable volumes of biological fluids. For example, TFF was used to process cell culture media (0.2 L) and lipoaspirate (1 L) and compared side-by-side with UC. The results demonstrated that TFF outperformed UC in yield, removal of single macromolecules and aggregates <15 nm and batch-to-batch consistency 57.

## Size-exclusion chromatography

SEC, also called gel filtration, allows to separate molecules varying in their hydrodynamic radius by passing them through a column gel. During the process, different molecular weight substances can penetrate the pores with different efficiency in the stationary phase of chromatography. Accordingly, the specific Stokes' radius vesicles in the mobile phase would segregate *via* the porous matrix according to their eluting time. Larger particles could rapidly pass through the column, whereas smaller particles come out in later fractions. Due to this property, SEC is available to separate EVs from blood plasma and urine samples 25, 61, 69. Critical impurities that may be hard to be separated by other methods in clinical samples such as soluble proteins and HDL can be mostly eluted from EVs by SEC 62. Commercial columns, in particular, qEV, Sepharose 2B, Sepharose CL- 2B and Sepharose CL-4B, have been developed to make the procedure easier. Although low numbers of lipoproteins are still visible, EV fraction isolated by SEC displays a low content of non-EV proteins 61. Moreover, researchers have shown EVs isolated by SEC display a high level of EV markers (which usually indicate a higher purity) as compared with UC 59, 60. Notably, Kawakami *et al.* 58 reported that EVs isolated from the serum of PCa patients using SEC have a significantly higher level of gamma-glutamyltransferase (GGT) than BPH patients, and thus EVs with GGT isolated from SEC could be used to diagnose PCa. SEC is considered faster and much more cost-efficient than UC. Therefore, this approach is an efficient method for isolation of EVs from small volume samples, especially from human plasma or serum when the sample volume is quite limited.

## Affinity interactions

As specific proteins/lipids are exposed on the surface of the EVs, affinity interactions can be used to capture EVs by binding to their surface receptors. For instance, antibody binding to EV receptors is an attractive way to isolate specific types of EVs. This immunoaffinity method can be approached by covalently binding antibodies to a stationary phase, such as surface of beads, paper-based (cellulose) filters, membrane affinity column and microarray slides 74-77. When biological fluids (e.g. plasma, serum, urine etc.) passing through the affinity surface, EVs would be captured *via* the binding between specific membrane markers (e.g. CD9, CD63, CD81, etc.) and the antibodies. This makes affinity binding outperform other methods in terms of selectivity and specificity as most non-membrane-based contaminants are removed. Affinity interactions can be also combined with other methods to make the isolation more efficient. For example, Mariantonia *et al*. 24 reported that immunocapture enzyme-linked immunosorbent assays (ELISA) assay can be combined with UC for capturing and analysing EVs from a PCa cell line and human plasma. Both CD81 and PSA were significantly elevated in PCa patients compared to BPH patients and healthy control subjects. Zhao *et al.* 78 combined immunoaffinity beads with microfluidic chip for isolating ovarian cancer plasma EVs and demonstrated that it can be used as a multiplexed measurement to diagnose ovarian cancer efficiently. Koliha *et al*. 63 introduced a multiplex beads platform to investigate 39 surface markers in one sample. The platform is based on polystyrene beads with the same diameter but labelling with varying amounts of 2 dyes to generate 39 distinguishable beads population in flow cytometry (FCM). The platform was used to analyse heterogeneous EV mixtures from natural killer (NK) cells, B cells and platelets. The findings suggested that the multiplex beads detection platform with different capture and detection antibodies can bring additional dimension for analysis compared to normal glass slides capture or protein microarray techniques. Recently, Chen *et al.* 79 reported using anion-exchange based beads to selectively separate EVs from PCa plasma with higher recovery efficiency (> 90%) and less protein contamination compared to UC. Affinity-based capture method yields highly enriched EVs away from non-membrane particle contamination and is simple and rapid without morphological change. Captured EVs are well prepared for some downstream analysis. For instance, EVs bound to microbeads can be used directly for FCM analysis and the captured EVs can be lysis immediately for protein or nucleic acid analysis. Although, for some downstream applications, such as drug delivery, intact EVs are required and thus further steps may be needed to detach EVs from the affinity interaction surface. Furthermore, the usage of reagents such as antibodies and beads increases the costs of this method. Although the common biomarkers such as CD63, CD81 and CD9 are widely used for EV capturing, it is acknowledged that there are currently no “universal” markers for all types of EVs 80. Thus, important EV subpopulations without the common biomarkers may be underestimated. Overall, affinity interaction method can be used in studying the heterogeneity natures of different subtypes of EVs, quantification of multiple analytes in a single sample, and provide important fundamentals for choosing targets for certain analytic tools such as biosensors.

## Microfluidic devices and microchips

The isolation methods aforementioned are mostly used for high-throughput but often require long processing time and bulky sample volumes which may bring extra stress to the patients. Recently, various methods have been developed for or applied on EV processing, aiming at reducing the sample volume and processing time and combining all isolation and analysis procedures into one lab-on-a-chip device. These methods are mainly based on microfluidic devices and chips. The microfluidic device is designed based on microscaled EVs isolation with manipulation of fluids through micro-channels. This approach processes with extremely small (10^-9^ to 10^-18^ litres) volumes of fluids compared to other isolation methods and is capable of handling viscous media in multiple channels 81. Besides, immunoaffinity beads, microporous filtration system 64, ultrasound purification 65 and dielectrophoretic enrichment (DEP) 66 have also been integrated for microfluidic devices and microchips for EVs isolation. Here we list a couple of very recently developed models which show great potential to be utilised in clinical settings.

Lewis *et al*. 66 recently used alternating current to fractionate pancreatic cancer EVs from serum and plasma samples on a nanoscale chip. The separation relies on alternating current electrokinetic associated with an immunoassay procedure. An alternating current electric field generated a DEP separation force, applied to particles in the fluids. Nanoparticles were trapped to the DEP high-field regions around the circular microelectrode, whereas larger entities remained in the DEP low-field regions and flushed away with the fluid. This method is very attractive as it does not require pre-treatment or dilution of the sample which is necessary for most of the other isolation methods. Like many other microfluidic devices, this method has advantages such as small sample volume (25-50 μL) and short processing time (30 minutes in total). Nonetheless, other types of particles in the range of 20-500 nm such as low-density lipoproteins (LDL) cannot be removed by this method. Also, high electrohydrodynamic force may generate heat dissipation in the medium which may change the natural states of the biological fluids.

**Figure 3:**
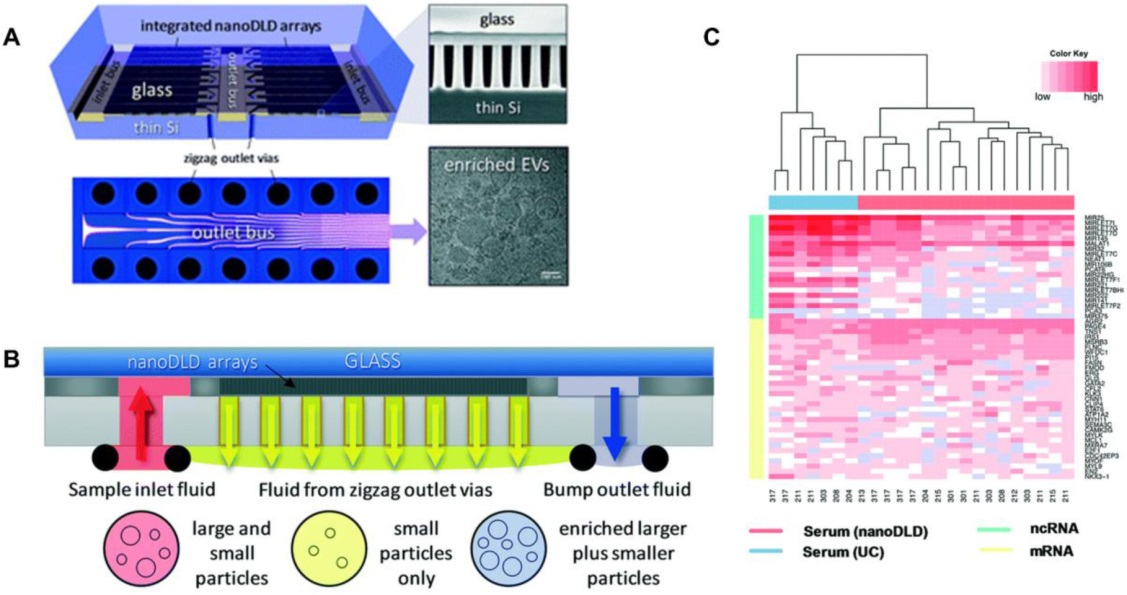
Integrated nanoDLD arrays for PCa EVs isolation and analysis. (A) Layout of integrated nanoDLD chip. (B) The workflow of sample injection with zigzag and enriched, bump-particle fluid isolation. Collection in common reservoirs on the back side of the chip from the different via sets. (C) Heatmap of 50 literature-curated PCa ncRNA and mRNA markers expression levels in EVs isolated from serum by nanoDLD and UC. Adapted with permission from 82, copyright 2018 Royal Society of Chemistry.

Smith *et al*. 82 reported an integrated nanoDLD arrays to isolate EVs (**Figure 3**). They demonstrated that this approach has a superior yield, smaller input and enhanced EV concentration compared to UC, UC with density gradient, qEV SEC (Izon Science), and the exoEasy Maxi Kit (QIAGEN) from both serum and urine samples. The integrated nanoDLD arrays are composed of 1024 parallel pillar arrays on a single chip to fractionate smaller particles and larger particles with zigzag and bump modes respectively (**Figure 3B**). Markers such as TSG101 and calnexin were used to identify EVs. They utilised RNA sequencing to profile small RNAs presented in EVs and reported the 50 most abundant PCa markers they found on RNA analysis (**Figure 3C**). Their results showed an improved gene expression correlation compared to UC. Thus, this method is a simple, high throughput approach which has great potential for PCa diagnosis and the discovery of EV-based proteins and RNA markers.

**Figure 4:**
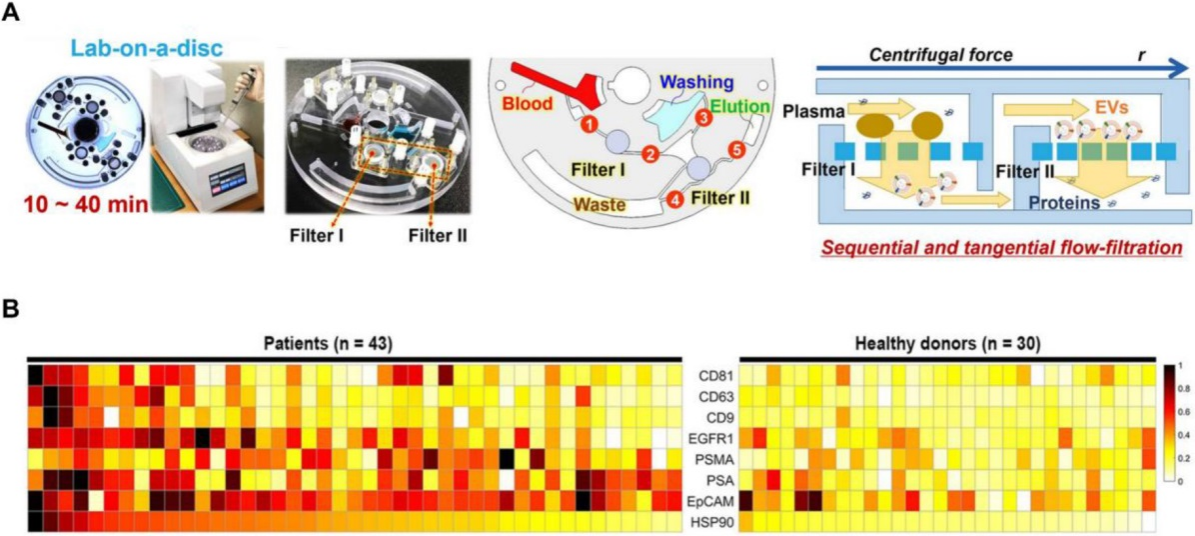
Exodisc for PCa EVs isolation. (A) Layout and working principle of Exodisc-B with centrifugal double-filtration system. (B) Heat map of protein types of human-plasma-driven EVs extracted from PCa patients and healthy donors. Measurements sorting along HSP90 expression level for three EV markers (CD81, CD63 and CD9) and five cancer markers (EGFR1, PSMA, PSA, EpCAM and HSP90) measured from lysates of EVs using direct ELISA. Adapted with permission from 83, copyright 2019 Ivyspring International Publisher.

Sunkara *et al*. 83 showed a fully integrated centrifugal microfluidic device and optimised it for EV isolation from whole-blood and plasma samples, respectively (Exodisc B and Exodisc P). This platform is operated with the sequential, tangential flow-filtration on a spinning disc with optimised filter membrane and spinning condition (**Figure 4A**). The isolated EVs were lysed and measured by ELISA, and significant differences of optical density were observed between PCa and healthy donors among PSA, PSMA, epithelial cell adhesion molecule (EpCAM), heat shock protein 90 (HSP90) and epidermal growth factor receptor 1 (EGFR1) (**Figure 4B**). Furthermore, this study also indicated that a combination of CD63, PSA, PSMA and HSP90 showed a higher sensitivity compared to HSP90 alone using support vector machine analysis. This platform has the potential to be used as a point-of-care system for early diagnosis of PCa as it is fast and fully automated with a higher yield and purity of EVs.

Overall, microfluidic device and chip-based methods have the potentials as low-cost, point-of-care devices for personalised cancer diagnosis. However, they are still in an early stage of development and have many disadvantages such as lower throughput, shear stress applied to the EVs, as well as mixing and clogging issues. And most importantly, the purity of the isolated EVs has not been properly or robustly validated for most of these methods. These issues should be addressed before microfluidic devices and microchips become practical in clinical settings.

## Analysis of EVs from prostate cancer samples

Quantitative and qualitative analysis of the EVs with specific cargoes is indispensable for different biofluids. Among the analytic methods, electron microscopy (EM) is used to characterize particle morphology; as an important method to study structural biology, cryo-EM can be used to observe natural state of EVs with a clear lipid bilayer. Cryo-EM can also differentiate EVs from dense particles such as lipoproteins and gives a direct understanding of sample purity 84 . Nanoparticle tracking analysis and resistive pulse sensing are used to characterize particle size distribution and concentration; however, they cannot differentiate EVs from contamination particles. Conventional protein analysis techniques such as western blots, ELISA and FCM can be used to determine the level of protein expression of EV components. Furthermore, the techniques for analysing nucleic acids such as RNA-sequencing, microarray, RT-qPCR, digital droplet PCR and PCR arrays can be used for EV RNA analysis.

**Figure 5:**
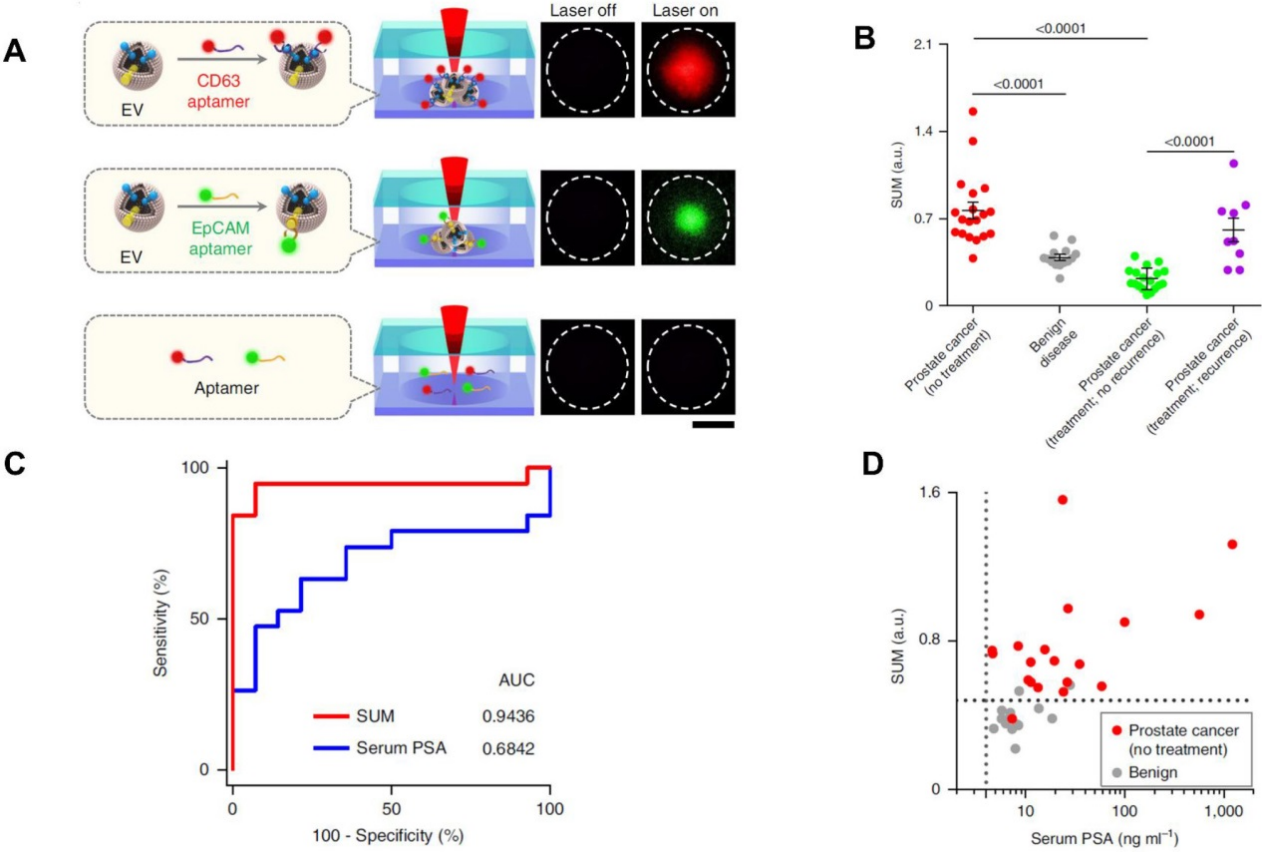
TAS for PCa EVs isolation. (A) EVs bound to aptamers detected by TAS. (B) The unweighted sum of the expression level of 7 markers (SUM signature) from EVs in different cohorts (means ± s.d.). (C) ROC curves showing a superior of SUM signature with higher accuracy (AUC = 0.9436) than serum PSA (AUC = 0.6842) in PCa versus benign disease discrimination. (D) Comparison of the SUM signature above 0.48 with serum PSA above 4 for the detection of PCa and benign disease. The vertical line indicates the threshold value of serum PSA (4 ng/mL) and the horizontal line indicates the threshold value of the SUM signature of EVs (0.48). Statistical differences were determined by two-tailed Mann-Whitney U-test. P values are indicated on the chart. Adapted with permission from 85. Copyright 2019 Springer Nature.

Recently, some novel analytic methods combined with separation methods have been applied in the PCa diagnosis. For example, Liu *et al*. recently developed a thermophoretic aptasensor (TAS) 85 to enrich EVs from serum, and further detected and classified 6 cancer types including PCa. This TAS used a panel of 7 aptamers conjugating EVs surface proteins to amplify signals. The amplified fluorescence signals give a profile of EV signatures after thermophoretic concentration (**Figure 5A**). By using TAS, several surface proteins including CD63, PTK7, EpCAM, LZH8, HER2, PSA and CA25 were investigated. All markers demonstrated an elevated level from PCa patients compared to healthy controls, except PSA. This evidence further confirmed that the current PSA marker is not accurate for PCa diagnosis or as an indicator of biologic aggressiveness. As shown in **Figure 5B,** a panel of the 7 protein markers can be used to distinguish PCa from BPH as well as recurrence from no recurrence. Furthermore, the results showed that the panel marker signature is superior to the PSA level detected from patients undergoing prostate biopsies (**Figure 5C and D**). TAS can be used to handle a crude serum sample in 3 hours and costs around US$1 for detection of 7 EV protein markers. Thereby, TAS can be implemented into early cancer screening as a low-cost, non-invasive assay. These novel approaches may inspire further progress in future EV-based PCa diagnosis.

In summary, each of the isolation methods has its own pros and cons (**Table 1**). There is currently no perfect method for all EV applications, and which isolation method to use depends on the downstream application. Currently, combination of different separation methods (e.g. SEC combined with affinity interactions or precipitation combined with UF) become more promising for a higher recovery and specificity compared to single type of isolation methods. Some of the co-separation particles may not affect the research if the target focuses on all EVs or other particular components but not a specific EV subtype. However, isolating pure EV subtypes would satisfy the requirement of tremendously increased reproducibility, make less confusing and bring consistent outcomes in functions, thus, further discover the actual target of choice for diagnostic applications. Due to the heterogeneity of EVs 40, 86, isolation and analytical techniques for different subpopulations can prompt the specificity and sensitivity of EV-based bioassay in clinical settings. Therefore, we believe further investigation is deserved on developing novel, specific and preferable panels of biomarkers for EVs and their specific subpopulations for different cancer subtypes.

## Potential EV protein biomarkers in prostate cancer clinical diagnosis

Plasma, serum as well as urine are commonly used for clinical studies of EVs and their specific cargos such as proteins, mRNAs, miRNAs, lncRNAs and lipids. As a major cargo, protein plays a significant role in functionalizing EVs. In recent years, due to a deeper insight into EV biogenesis pathways, studies showing that protein constitution of EVs are indicative of epithelial-mesenchymal transition and carcinogenic characteristics 87-89. Depending on the specific clinical questions, i.e. whether it is for diagnosis, prognosis or treatment monitoring, the PCa EVs biomarker targets could be different. Understanding the different types of EVs can reveal functional activities and translate the findings into clinical application. The complex biomolecules (specific proteins) associated with different types of EVs are considered as a precious target for PCa early diagnosis.

Unfortunately, little is known about the EV-carried proteins and a consensus has not yet emerged on specific markers of EV subtypes 33. Worse still, due to the heterogeneity of EVs, no universal markers apply to all types of EVs. Accordingly, International Society for Extracellular Vesicles recently summarised five categories of proteins, in order to help researchers demonstrate the presence of EVs as well as the purity of EVs or its subtypes. The five categories include three major categories that must be analysed and two additional categories for specific EV subtypes and the functional study 33. Proteins of Category 1, as one major category, including transmembrane or Glycosylphosphatidylinositol-anchored proteins associated to plasma membrane and/or endosomes, should be used along with another type of “positive control” protein markers (Category 2) and a type of “negative control” (Category 3) to confirm the presence of EVs. Tetraspanins CD9, CD63 and CD81 are the most common surface protein markers to demonstrate the presents of lipid-bilayer structure in any type of EVs. Cytosolic proteins such as ESCRT-I/II/III (e.g. TSG101) and Flotillins-1 and 2, are also required to be analysed in order to demonstrate the enclosing of intracellular material. Furthermore, components of non-EV co-isolated structure such as lipoproteins and albumin should be analysed as well, which belongs to the “negative control”. If the specificity of EV subtypes or EV-associated receptor are studied, proteins associated with intracellular compartments other than plasma membrane/endosomes or secreted or luminal proteins that bind to specific receptors on the EV surface should be analysed as well.

Fortunately, various types of proteins, such as plasma membrane surface proteins and cytosolic proteins, are associated with cancer-derived EVs with parent cell information. Indeed, it has been indicated that proteins on the EV surface are involved in cancer development. For example, EVs derived from metastatic melanomas carry programmed death-ligand 1 (PD-L1) on the surface, which inhibits the function of CD8 T cells and facilitates tumour growth 90. Similarly, protein surface markers on PCa-derived EVs may also be associated with PCa progression 91, 92. Multiple biomarkers will likely be required to cover different clinical questions. Since EVs is a promising candidate for liquid biopsy, reports to summarize EV-based PCa diagnosis researches are in great demand. Here, we summarized potential protein-based biomarkers of PCa-derived EVs for screening and diagnosis purposes that have been identified so far in **Table 2**. For other types of biomarkers such as nucleic acid and lipid-based markers, the readers are referred to other review papers for these topics 35, 93, 94, which are out of scope of this review.

Modern “-omics” techniques including high-throughput proteomics (e.g. mass spectrometry) and genomics (e.g. next-generation sequencing) have been used to identify promising PCa biomarker candidates from cell lines, blood and urine. Proteomic analysis of EVs from PCa cell lines and cancer patients' samples in liquid biopsy showed that EVs are a rich source of intracellular proteins. Minciacchi *et al.* 95 showed that cytokeratin 18 (CK18) was significantly enriched in EVs from PCa plasma which was in line with PCa tissue identification. Serum exosomal GGT activity was detected by a newly reported fluorescence probe, gGlu-HMRG, and found significantly higher in PCa patients compared with BPH patients. The AUC of serum exosomal GGT activity was the highest among serum GGT activity, serum PSA concentration and itself 58, suggesting the GGT may be used as a serum exosomal marker to separate PCa and BPH with close PSA levels. Similarly, CD9, CD81, CD63 positive EVs from PCa urine samples have a significantly overexpressed level of fatty acid binding protein 5 (FABP5) associated with Gleason score compared to the healthy control group 101. Øverbye *et al.* 102 found protein markers transmembrane protein 256 (TMEM256) and late endosomal/lysosomal adaptor, MAPK and MTOR activator 1 (LAMTOR1) were enriched in PCa urine sample. The combination of the two makers can reach an AUC= 0.94, suggesting the advantage for multiplexing biomarkers. Protein markers V-type proton ATPase 16 kDa proteolipid subunit (VATL), adipogenesis regulatory factor (ADIRF), and several Rab-class markers were also enriched in the PCa EVs. However, author didn't give many deep discussions of these three markers which can be further investigated. Wang *et al.* 103 demonstrated that exosomal proteins of flotillin 2 and parkinsonism associated deglycase (PARK7) from PCa urine EVs can be considered as a potential multimarker panel in clinical settings. Similarly, it was reported that different protein marker panels in urine EVs can be used for different enpoint questions. For instance, the combination of Adseverin and Transglutaminase-4 can distinguish BPH and PCa patients; while the combination of CD63, Putative glycerol kinase 5, N-sulphoglucosamine sulphohydrolase, PSA, Prostatic acid phosphatase can separate high- and low-grade PCa^104^. Bijnsdorp *et al.* 105 demonstrated exosomal proteins Integrin subunit alpha 3 (ITGA3) and Integrin subunit beta 1 (ITGB1) were enriched in urine samples from metastatic patients compared to those from BPH or PCa patients.

Apart from identifying novel EV diagnostic biomarkers using proteomics, it is also promising to investigate whether those reported biomarkers in blood or tissue have an outstanding performance on EVs, due to EV direct pathway from tumour and with less interference of same protein from different source as a multiparameter tool. As mentioned above, the diagnostic performance of serum PSA is modest in terms of specificity, however, PSA from EVs has shown great potential as a more reliable marker. Logozzi *et al.* 24 recently showed that PCa patients had a four-fold more nanovesicles expressing both CD81 and PSA from plasma compared to BPH patients and healthy controls by quantifying their levels using nanoscale FCM and ELISA. Also, their results showed that the tumour microenvironmental acidity pressure selectively stimulated the release of nanovesicles and PSA expression from a human PCa cell line. 101. Øverbye *et al.* 102 also showed that well known PCa protein markers PSA, FOLH1/ PMSA, TGM4, and TMPRSS have a higher expression in EVs derived from PCa patients compared to normal controls. Phosphatase and tensin homolog (PTEN) is a potent tumour-suppressor protein in blood. Reduction in PTEN expression supports the invasion and metastatic behaviour of PCa 106. It was found that PTEN secreted in the exosomes could be internalised by the recipient cells, with resultant inactivation of phosphatidylinositol 3-kinase/AKT pathway and associated reduced cell proliferation 107 . Interestingly, Gabriel *et al*. 96 demonstrated that PTEN was expressed in PCa-derived exosomes but not in normal cell-derived exosomes, even though normal cells themselves express PTEN. These studies suggested that EVs play an important role in transferring PTEN beyond cells, and implicated an exclusionary mechanism used by PCa cells to downregulate PTEN, possibly by selective packaging. However, due to the limited sample size in the study 96, further validation and investigation of the responsible molecules are needed. Park *et al*. 97 found that PSMA and CD63 positive EVs had a different expression between BPH and PCa patients, which was consistent with the pathologic outcomes. Khan *et al*. 98 reported that Survivin from serum/plasma-derived exosomes has a higher expression in patients with PCa compared to BPH and healthy controls. It suggests that Survivin is a promising research candidate in comparing patients with and without tumours, but both with high PSA values. This could be used to more accurately differentiate BPH from PCa in the early diagnosis. P-glycoprotein (P-gp) has been reported to be related to resistance against chemotherapy for castration-resistant PCa (CRPC) patients 108. Kato *et al*. 99 demonstrated that P-gp had a higher expression in exosomes released from docetaxel-resistant PC-3 cells. Furthermore, they found p-gp in PCa patients' blood exosomes participate in docetaxel-resistance other than cabazitaxel-resistance, indicating exosomal p-gp could be used in diagnose docetaxel-resistant patient and choose a proper taxoid. δ-Catenin was found in brain and upregulated in PCa tissues 109. Lu *et al*. 100 demonstrated that δ-catenin, caveolin-1, and CD59 were positive in human PCa urine EVs, indicating these markers can be used for PCa detection. All these data suggest that tumour-derived EVs carrying specific protein markers are potentially promising tools to improve screening/diagnosis of PCa.

Glycan sugar groups, closely associated with PCa development and progression, are an interesting research area and holding promise in stratification of PCa patients through multi-omic platforms. For example, there are about 50 glycoforms of PSA that have been investigated and only some of them were found in aggressive PCa, especially glycoforms with α2,3-sialic acid 110. Therefore, it is generally recognized that the status of the various classes of glycosylation and glycoprotein promotes cancer development and can be used for PCa diagnosis and therapeutics 111, 112. It was reported the different functions of EV proteins are correlated with the carbohydrate structures conjugated to it as glycans or as repeating glycosaminoglycan chains in proteoglycans 112-114. Aberrant glycosylation is highly associated with the protein functions in cancer and can be used as a hallmark of cancer 112, 115.

Because of the enrichment of specific glycans to EV proteins, glycomics can be used to study EV surface glycans or glycoproteins such as prostatic acid phosphatase (PAP) and PSA to improve PCa diagnosis 111, 112. For example, N-linked glycan of exosomes derived from non-cancer and PCa patients of expressed prostatic secretions urine samples analysed by mass spectrometry showed a higher proportion of tetra‐antennary glycans in the aggressive PCa exosome samples. A clear difference was also found between cancer samples and non-cancer samples particularly at 2271 m/z which are consistent with a bisecting GlcNAc‐hybrid structure of Gal1N2M5N2 116. Several pilot studies from metastatic PCa serum EVs isolated with ExoQuick kit also showed common glycans yield in both total glycoprotein fractions and the exosome fraction, and multiple glycans specific to either fraction, indicating that the common and distinct species can be applied to screening assays of liquid biopsy sample cohorts 117. However, some of the issues such as insufficient EVs, EV isolation modification, inappropriate blocking agents and contaminations may need to be further addressed regarding EV glycans studies. For example, urine samples usually accompanied with Tamm-Horsfall proteins can form aggregates. Due to the high glycosylation, a prior removing of this kind of proteins is required 118. Although not much detail is known about how glycosylation is functionally responsible for EVs in cancer biology and glycan of EVs in PCa is largely unexplored, a better understanding of glycans and glycoproteins associated with EVs may provide a new avenue for PCa diagnosis and progression monitoring.

In summary, among EV protein biomarkers in PCa, several researchers have shown that quantitative assay of a combination of multiple EV-derived proteins might enhance the diagnostic efficacy compared to using a single serum-derived protein marker such as PSA. Because of the heterogeneity of EVs among different subtypes and different patients, a single marker may be lack of power to reflect the multifactorial essence of PCa, nor does it have the satisfactory discriminative precision for accurate risk-classification 102, 104, 119. Therefore, further investigation is warranted to discover, develop, then internally and externally validate panels which combine multiple biomarkers from tumour-derived EVs. Furthermore, according to the guideline from International Society for Extracellular Vesicles, more EV markers should be investigated to associate with EV subtypes and to track their origins and pathways involved, illustrating the purity and specificity of EV samples further.

## Current challenges for EV studies using blood samples and applications in prostate cancer

As an abundant and easily extractable source of body fluid for liquid biopsy, blood is highly associated with EV study and further clinical applications. Injured, stressed or diseased cells such as tumour cells behave self-reaction and release molecules into the bloodstream. Since EVs are directly derived from their parental cells, exploring whether they can be used as a provider of potential cancer biomarkers is critical for cancer research. Clinical studies of EVs confirmed that EVs from blood of PCa patients are extremely abundant compared to BPH patients or healthy volunteers 24, 97. Specially, Tavoosidana *et al*. 37 reported an elevated exosomal level with PCa in blood (median 7.7 ng/mL; range 1.1-34.9 ng/mL) compared with matched controls (median 1.1 ng/mL; range <1.1-12.4 ng/mL). Investigation of tumour-derived EVs as the biomarker in blood holds promise, whereas it also faces great challenges largely because of the nanoparticle's complexity and dynamic fluctuation of their concentration in peripheral blood. Lipoproteins are assembled with cholesterol esters and triglycerides in the centre surrounded by free cholesterol, phospholipids, and apolipoproteins. Plasma/serum lipoproteins have very close buoyant density and physical size compared to the EVs and are abundant in the circulation. For example, Chylomicrons are a type of lipoprotein formed in the intestinal epithelium with particle size ranging from 75 to 1200 nm in diameter; very low-density lipoproteins (VLDL) are smaller, with a particle range from 30-80 nm, and the smallest three lipo-protein subtypes - intermediate-density lipoprotein (IDL), LDL and HDL - have a particle size of 5-35 nm 120. Previous researchers 31, 61, 121 showed that EVs segregated from plasma are over 100-fold less prevalent than lipoproteins using UC and most of the contaminants are LDL and HDL. SEC achieves lower lipoprotein contamination than UC, but still has the issue of contamination with LDL 122. Moreover, albumin and immunoglobulins are proteins largely existing in the blood and can also be co-isolated with EVs with traditional separation methods 123. Such contaminants can mask the desired EVs marker signal in the investigation, thus, can be set as purity control for the EVs processing. For current research, we recommend the combination of distinct separation techniques, such as SEC combined with immunocapture, for isolating EVs distinctly from lipoprotein and albumin.

In addition, previous studies have shown that EV performance was sensitive to pre-analytical variables. During blood sample handling, artificial elevation of EVs matters as a result of platelet activation, which may increase platelet-EVs release, due to blood collection and transportation as well as unnecessary delays in plasma/serum separation 124. Proper guidelines, such as International Organization for Standardization standard 15189 assays or those from International Society on Thrombosis and Haemostasis, should be applied to the processing including the interpretation of test results, proper tubes for blood collection, and sufficient separation of platelet-free plasma using multiple rounds of centrifugations immediately after blood collection. Studies have shown that sample handling with EDTA tubes may provide better stability of EV counts in samples than heparin and sodium citrate tubes 125. It has been also shown that two-step centrifugation benefits EDTA plasma samples with lower background intensity from microarray analysis 126. However, it may be difficult to apply rigorous blood collection and plasma separation/storage in a large-scale clinical setting. Thereby, Minimal Information for Studies of EVs 2018 suggests recording as many pre-analytical parameters as possible 33. Furthermore, other non-relevant diseases and medical therapy, such as hormone therapy, may significantly affect the EVs release which influences EV-based diagnosis results. Thus, blood sample collection as well as grouping should be properly designed for clinical studies.

## Conclusions

PCa significantly affects men's health, and circulating EVs show promise as a key player in liquid biopsy. EVs play a significant role in transferring cellular signals to recipient cells under both physiological and pathological conditions. Investigating markers within EV cargos for clinical studies could be extremely valuable in discovering EVs as novel biomarkers for better diagnosis in PCa. In conclusion, this review summarises promising isolation methods that could promote the isolation and analysis of EVs in the clinical setting, including conventional methods and novel methods, and discusses the advantages and drawbacks of each. Cancer researchers should choose suitable isolation and analytic methods based on their own parameters such as original EV media, sample size, downstream analysis. We also summarised current EV-based protein biomarkers that have been identified for PCa from both cell lines and human samples such as blood and urine, which could substantially be used as a guide to PCa EVs biomarker investigation. Cancer-derived EV markers from various biofluids are valuable markers and can be taken a step further to correlate with EVs and PCa clinically especially in different stages for diagnostic purpose. Finally, we discussed several major hurdles that EV researchers may need to pay attention to before EVs can be applied in the clinic. Following studies can focus on these hurdles and avoid inappropriate handling that may affect EVs.

For PCa diagnosis, it is crucial to choose proper isolation methods and analysis techniques as different methods yield different purity and subtypes of EVs. Different subtypes of EVs carry various information and may result in a conflict result for PCa diagnosis. Thus, there is a demand to instigate how EVs perform differently among different isolation and analysis methods. Such researches would largely improve both process and accuracy using EVs as diagnostic tool and promote the conversion of EV studies to the clinical, further benefit the clinical decision-making. Our review can serve as a basis for further investigation in PCa related EVs biomarker discovery. We believe that EVs hold a bright future in PCa assessment and can better-improve diagnosis and reduce cancer health disparities.

## Figures and Tables

**Table 1:** Summary of common EV isolation approaches in PCa

Isolation Method	Mechanism	Advantage	Limitation	Reference
**Ultracentrifugation and density gradient**	Mass and density	Large sample capacity, low following cost, low background contamination (with density gradient-based approach)	Low efficiency for small sample volume, high capital cost, time-consuming, unexpected aggregation, protein lost	25, 31, 40-44
**Precipitation**	Surface charge or solubility change	Very easy handling, scalable, does not deform EVs	High background contamination for complex component sample (e.g. blood). Chemicals (polyethylene glycols or similar) used may impair downstream analysis	42, 45-51
**Ultrafiltration**	Size	Cut-off specific particle size, fast, less deformation of EVs	Limited filter lifetime, extra cleaning step, extra force, protein contamination	25, 52-54
**Field-flow fractionation**	Size and molecular weight	Continuous operation, fraction population for further analysis	Extra force applied to the field, protein contamination	55-57
**Size-exclusion chromatography**	Size and molecular weight	No extra force involved, does not deform EVs, can remove high-density lipoprotein (HDL)	Contamination from particles with similar size	58-62
**Affinity interactions**	Affinity binding	Specific interaction to the target, high purity	Pre-purification or combination steps may be needed, not for large scale	24, 63
**Microfluid and microchips**	Size, density or affinity binding	Low sample amount, fast, isolation and analysis can be integrated	Not suitable for large scale, specific design required, low EV yield	64-66

**Table 2:** Summary of EV protein biomarkers identified in PCa for clinical diagnosis

Putative PCa marker	Human sample	EV source	Markers for EV identification	EV isolation	Stage of the disease	Main result	Ref.
CK18	PCa patients (n=6), healthy controls (n=5)	Plasma, cell line (DU145)	CD81, TSG101	UC; Precipitation	N/A	CK18 were significantly enriched in EVs and in line with CK18 IHC in human PCa tissues	95
PSA	PCa patients (n=15), BHP patients (n=15), healthy controls (n=15)	Plasma, cell line (LNCaP)	CD81, TSG101	UC	Prostate adenocarcinoma, total PSA was > 2.5 ng/ml	LNCaP EVs express significant levels of PSA PCa had four-fold higher PSA-specific exosomes than BPH and healthy controls Acidic condition stimulated the release of PSA-specific exosome	24
PTEN	PCa patients (n=30), healthy controls (n=8)	Plasma, cell lines (DU145, PC-3, U87, HAOEC, HAOSMC, HPEC)	Flotilin-1	UC	Advanced (T3/T4) tumour stage	PTEN was detected in PCa cell/plasma exosomes instead of normal cell/plasma exosomes	96
PSMA	PCa patients (n=82), BPH patients (n=28)	Plasma	CD63	Precipitation	Low- (n = 17); intermediate- (n = 36); high-risk (n = 29) PCa according to the National Comprehensive Cancer Network risk group	PSMA EVs concentration was higher in PCa than BPH Patients with lower PSMA EVs concentration had a larger prostate volume, lower GS and lower risk of biochemical failure	97
Survivin	PCa patients (n=39), BPH patients (n=20), healthy controls (n=16)	Plasma; serum	Lamp1	UC; Precipitation	Plasma: ten low-grade PCa cases (GS 6); ten high-grade PCa cases (GS 9); Nineteen serum samples: N/A	Survivin existed in plasma exosomes of normal, BPH and PCa Survivin level in PCa exosome was significantly higher than BPH and Control Survivin exosome level in relapsed chemotherapy patients was higher than controls	98
GGT	PCa patients (n=31), BPH patients (n=8)	Serum, cell lines (LNCaP, C4, C4-2, C4-2B)	CD9, PDCD6IP	UC, Size-Exclusion Chromatography, Immunoaffinity intonations	PCa patients: PSA: 4.20-28.23 ng/mL BPH patients: 4.42-25.40 ng/mL	GGT1 was elevated in exosomes isolated from C4-2 and C4-2B cells The level of GGT-specific exosomes was significantly higher in PCa than BPH	58
P-gp	CRPC patients (6 therapy-naïve and 4 clinically docetaxel-resistant PCa patients)	Serum, cell lines (PC-3, PC3-R)	CD9	UC	6 therapy-naïve: PSA: 1.481-7.875 ng/mL, GS 6-8; 4 clinically docetaxel resistant PCa patients: PSA: 26.177-25313.0 ng/mL, GS 8-10	P-gp was higher in PC-3R exosomes than PC-3 P-gp exosome level was higher in clinically docetaxel-resistant patients than in therapy-naïve patients	99
δ-Catenin, caveolin-1, CD59	PCa patients (n=16) PCa inactive patients (n=15)	Urine, Cell lines (PC-3, CWR22Rv-1)		UC	PSA: 0.3-667 ng/mL, GS 6-8	δ-Catenin, caveolin-1, CD59 were detected in cell-free urine EVs δ-Catenin had a significant increase in PCa compared to PCa inactive patients	100
FABP5	PCa patients (n=30), negative control group* (n=17)	Urine, cell lines (PC3, DU145)	CD9, CD63, CD81	UC	GS6, PSA: 4.1-126 (n=6); GS8-9, PSA: 6.8-311 (n=6); low-risk PCa, GS6, PSA: 2.9-10.6 (n=5); high-risk PCa, GS7-9, PSA: 4.3-3143 (n=13)	FABP5 was overexpressed in PCa EVs compared to negative control group FABP5 in urinary EVs was significantly associated with GS	101
TMEM256- LAMTOR1, ADIRF, VATL, Rab, PSA, FOLH1/ PMSA, TGM4, TMPRSS	PCa patients (n=17), healthy controls (n=15)	Urine	CD9, CD81, TSG101	UC	GS6-9, PSA: 4.5-23 (n=16); 1 patient N/A due to the low exosomal protein yield and excluded from the proteomic analysis	Combined TMEM256- LAMTOR1 augmented the sensitivity to 100% with AUC= 0.94 ADIRF, VATL and 18 different Rab proteins were enriched in PCa urinary exosomes PSA, FOLH1/PMSA, TGM4, TMPRSS were enriched in the urinary exosomes as well but with lower degree of specificity and /or sensitivity compared to TMEM256- LAMTOR1	102
PARK7- Flotillin 2	PCa patients (n=26), healthy controls (n=16)	Urine	N/A	UC	GS6-8, PSA: 4.4-22.6 (n=26)	Flotillin 1, Flotillin 2, Rab3B can be used to separate PCa and healthy males Flotillin 2 detected by Western blot has AUC-0.914 ELISA tests of Flotillin 2 and PARK7 can discriminate PCa from control with AUC of 0.65 and 0.71 respectively Combination of Flotillin 2 and PARK7 improved the diagnostic accuracy and robustness	103
ADSV-TGM4, CD63-GLPK5-SPHM-PSA-PAP	PCa patients (n=53), BPH patients (n=54)	Urine	CD81, TSG101, RaB5	UC	Low-grade PCa GS ≤ 7 (3+4) (n=22); high-grade PCa GS ≥ 7 (4+3) (n=31)	Combination of protein panel improved the ability to distinguish benign from PCa samples ADSV-TGM4 AUC=0.65 CD63-GLPK5-SPHM-PSA-PAP AUC=0.70	104
ITGA3, ITGB1	Metastatic PCa patients (n=3), PCa patients (n=5), BPH patients (n=5)	Urine, cell lines (LNCaP, PC3)	PDCD6IP	UC	Metastatic PCa patients (n=3); PCa patients (n=5),	ITGA3 showed low level in LNCaP exosomes and was hardly detectable in PC3 exosomes. ITGB1 was not detectable in LNCaP exosomes and had some expression in PC3 exosomes ITGA3, ITGB1 had a higher level in metastatic patient than BHP and PCa	105

**Notes:** *The “negative” group comprised the patients who received prostate biopsy due to elevated PSA levels and were diagnosed pathologically as negative. **Abbreviations**: ADIRF : adipogenesis regulatory factor; ADSV: Adseverin; AUC, Area under the curve; BPH: benign prostatic hyperplasia; PCa, prostate cancer; CD9: CD9 molecule; CD63: CD63 molecule; CD81: CD81 molecule; CK18, cytokeratin 18; CRPC, Castration-resistant prostate cancer; ELISA: enzyme-linked immunosorbent assays; EVs: Extracellular vesicles; FABP5: Fatty acid binding protein 5; GGT: Gamma-glutamyltransferase; GLPK5: Putative glycerol kinase 5; GS, Gleason score; IHC, Immunohistochemistry; ITGA3: Integrin subunit alpha 3; ITGB1: Integrin Subunit Beta 1; LAMTOR1: Late endosomal/lysosomal adaptor, MAPK and MTOR activator 1; N/A: not applicable; PARK7: Parkinsonism associated deglycase; PAP: Prostatic acid phosphatase; PDCD6IP: programmed cell death 6 interacting protein; P-gp: P-glycoprotein; PSA, Prostate specific antigen; PSMA, Prostate-specific membrane antigen; PTEN: Phosphatase and tensin homolog; RaB5: RAB5A, member RAS oncogene family; RAB3B: RAB3B, member RAS oncogene family; SPHM: N-sulphoglucosamine sulphohydrolase; TGM4: Transglutaminase-4; TMEM256: Transmembrane protein 256; TMPRSS: transmembrane protease, serine 2; TSG101: Tumour susceptibility gene 101; VATL: V-type proton ATPase 16 kDa proteolipid subunit

## Abbreviations

AF4: asymmetrical flow field-flow fractionation
AQP2: aquaporin-2
BPH: benign prostatic hyperplasia
PCa: prostate cancer
CK18: cytokeratin 18
CTCs: circulating tumour cells
cfDNA: cell-free DNA
DEP: dielectrophoretic enrichment
EGFR 1: epidermal growth factor receptor 1
ELISA: enzyme-linked immunosorbent assays
EM: electron microscopy
EpCAM: epithelial cellular adhesion molecule
EVs: extracellular vesicles
ExoTIC: exosome total isolation chip
FABP5: fatty acid binding protein 5
FCM: flow cytometry
FFF: field flow fractionation
GGT: gamma-glutamyltransferase
GLPK5: putative glycerol kinase 5
HSP90: heat shock protein 90
HDL: high-density lipoprotein
IDL: intermediate-density lipoprotein
LDL: low-density proteins
MWCO: molecular weight cut-off
nanoDLD: nanoscale deterministic lateral displacement
NSE: neuron-specific enolase
NK: natural killer
PAP: prostatic acid phosphatase
PARK7: parkinsonism associated deglycase
PDCD6IP: programmed cell death 6 interacting protein
PD-L1: programmed death-ligand 1
P-gp: p-glycoprotein
PODXL: podocalyxin like
PSA: prostate specific antigen
PSMA: prostate-specific membrane antigen
PTEN: phosphatase and tensin homolog
SEC: size-exclusion chromatography
SPHM: n-sulphoglucosamine sulphohydrolase
TAS: thermophoretic aptasensor
TFF: tangential flow filtration
TGM4: transglutaminase-4
TMEM256: transmembrane protein 256
TSG101: tumour susceptibility gene 101
UC: ultracentrifugation
UF: ultrafiltration
VLDL: very low-density lipoproteins
